# Musculoskeletal Disorder Risk Assessment during the Tennis Serve: Performance and Prevention

**DOI:** 10.3390/bioengineering11100974

**Published:** 2024-09-27

**Authors:** Philippe Gorce, Julien Jacquier-Bret

**Affiliations:** 1 International Institute of Biomechanics and Occupational Ergonomics, 83418 Hyères, France; gorce@univ-tln.fr; 2 University of Toulon, CS60584, 83041 Toulon, France

**Keywords:** biomechanics, optoelectronic system, 3D motion analysis, ergonomic assessment, REBA, tennis serve, performance, coaching

## Abstract

Addressing the risk of musculoskeletal disorders (MSDs) during a tennis serve is a challenge for both protecting athletes and maintaining performance. The aim of this study was to investigate the risk of MSD occurrence using the rapid whole-body assessment (REBA) ergonomic tool at each time step, using 3D kinematic analysis of joint angles for slow and fast serves. Two force platforms (750 Hz) and an optoelectronic system including 10 infrared cameras (150 Hz, 82 markers located on the whole body and on the racket) were used to capture the kinematics of the six REBA joint areas over five services in two young male and two young female ranked players. The mean REBA score was 9.66 ± 1.11 (ranging from 7.75 to 11.85) with the maximum value observed for the loading and cocking stage (REBA score > 11). The intermediate scores for each of the six joint areas ranged between 2 and 3 and the maximum value of their respective scales. The lowest scores were observed for the shoulder. Neck rotation and shoulder flexion are parameters that could be taken into account when analyzing performance in the context of MSD prevention.

## 1. Introduction

The tennis serve is a complex movement that must be mastered to gain an advantage over the opponent. Control of ball velocity and trajectory is conditioned by racket control, which is linked to the kinematics of the player’s body. In order to study the execution of a serve, many authors have divided it into phases based on key postures. Kovacs and Ellenbecker [1] proposed a three-phase decomposition with eight stages as follows: the preparation phase with four stages (start, release, loading, and cocking), the acceleration phase with two stages (acceleration and ball contact), and the follow-up phase with two stages (deceleration and finishing). Five key points of interest have been classically identified in the literature [1,2]. These are (1) the initial position with the racket at rest (start); (2) the ball release (BR) when the ball leaves the non-serving hand; (3) the trophy position (TP) with minimal vertical elbow position and maximum knee flexion; (4) the racket low point (RLP) when lateral shoulder rotation is maximal and the racket head is pointing downwards; and (5) the ball impact (BI).

To master this technique, a detailed knowledge of kinematics is needed to improve performance, often considered in terms of ball or racket velocity. These parameters are affected by several factors, such as service side, service type, or stance style, as many studies have demonstrated. For example, Reid et al. reported a difference in knee extension velocity as a function of stance style [3]. The foot-up technique (placing the back foot next to the foot before the jump) generates a greater knee extension velocity than the foot-back technique (keeping the feet offset, one forward and the other backward, until the jump [4]). Hornestam et al. [5] also reported that knee flexion had an impact on racket velocity. Comparing two groups with different flexions, the authors showed that the group with the lowest knee flexion generated a lower racket velocity than the group with the highest knee flexion. Reid et al. also showed that a kick serve led to a lower racket velocity than a flat serve in high-level players [6].

Kinematic analyses were also carried out at different key points, especially TP, RLP, and BI [7]. For TP, the authors mainly studied trunk position as well as knee and ankle flexions (front and back) as a function of several parameters. Trunk inclination and rotation, respectively, assessed from 17.0 ± 11.0° to 34.3 ± 7.6° and from 4.0 ± 10.0° to 27.3 ± 25.5°, were affected by age (children, teenagers, and adults [8]), level (expert vs. non-expert [9]), and stance style (foot-up vs. foot-back [3]). Knee and ankle flexions were analyzed as a function of sex [10,11], age [12], type of serve (flat, slice, and topspin serve [13]), and racket size [14] with values ranging between 47.0 ± 21.0° and 82.8 ± 12.8° for the knees and between 0.3 ± 22.3° and 19.8 ± 3.4° for the ankles. For these two joint angles, some authors compared the values obtained for the front and rear lower limbs [13,14,15]. A few studies reported values for the upper limb, notably, on shoulder axial rotation (from 60.0° to 76° [16,17]), elbow flexion (77.8 ± 35.1° to 107.0 ± 30.0°), and wrist flexion (2.0 ± 10.0° to 16.0 ± 11.0°) [9,12].

For RLP, the joint angle most studied in the literature has been shoulder lateral rotation, which is the parameter that defines this key point [1]. The value of shoulder lateral rotation has been measured as a function of multiple parameters such as the type of serve (flat: 89.8° [18]; kick: 119.0 ± 18.3° [6]), the side of serve (deuce: 136. 7 ± 10.6°; ad: 138.1 ± 11.4° [19]), fatigue condition (125.0° with and without fatigue [16]), and age (children: 152.0 ± 32° [2]; adults: 141.0 ± 7.0° [8]).

The player’s posture at the moment of ball impact has also been the subject of numerous studies under a variety of conditions. Shoulder abduction and elbow flexion were the two most commonly reported parameters in these studies. The results showed a slight elbow flexion at BI for the following conditions: sex (male 10.7 ± 6.6°; female: 34.7 ± 4.0° [10]), age (children: 44.0 ± 13. 0 [2]; adults: 27.0 ± 8.0° [8]), level (expert: 5.4 ± 7.8°; non-expert: 79.9 ± 4.9° [9]), and side of serve (deuce: 18.0 ± 7.8°; ad: 18.0 ± 8.5° [19]). Shoulder abduction was assessed at nearly 100° under the following conditions: sex (male 150.3 ± 4.9°; female: 161.1 ± 1.3° [10], age (children: 92.0 ± 9.0° [2]; adults: 104.0 ± 13.0° [8]), and side of serve (deuce: 114.0 ± 6.4°; ad: 114.5 ± 6.4° [19]). Trunk inclination (>25° [2,20]), wrist flexion (20 to 30° [11,19]), knee flexion (20 to 30° [19,21]), and ankle extension (approx. 40° [8,11]) have also been reported in some studies.

In conjunction with the kinematic analysis of the serve, the question of preventing musculoskeletal disorders (MSDs) and their consequences in tennis players has been addressed in the literature in a descriptive way. MSDs are defined by the World Health Organization as health problems of the locomotor apparatus, i.e., muscles, tendons, bone skeleton, cartilage, ligaments, and nerves. This includes any type of complaint, from slight transitory discomforts to irreversible and incapacitating injuries [22]. They can be caused by acute trauma (e.g., fractures, sports injuries), tissue degeneration (e.g., osteoarthritis, spinal stenosis), genetic aberrancies (e.g., muscular dystrophy), and autoimmunity (e.g., rheumatoid arthritis) [23]. Martin et al. compared differences in the onset time of several biomechanical events between a group of healthy and a group of injured players [24]. However, to our knowledge, no study has objectively quantified and qualified the level of risk when serving in tennis. There are many tools available to assess MSD risk. Gómez-Galán et al. proposed an exhaustive list of these tools and classified them into three groups as follows: direct, indirect, and semi-direct methods [25]. Semi-direct methods use posture evaluation grids and additional activity-related criteria to assess MSD risk. Among the 18 methods listed, the REBA—Rapid Entire Body Assessment [26] method enables the whole body to be taken into account in posture assessment, using angular value thresholds, unlike other methods such as RULA—Rapid Upper Limb Assessment [27], LUBA—postural loading on the upper body assessment [28], OWAS—Ovako Working Posture Analyzing System [29], and RAMP—Risk Assessment and Management tool for manual handling Proactively [30].

The aim of the present study was to evaluate tennis serve performance by integrating an MSD risk assessment using the REBA in order to prevent and better understand the onset of MSDs. Perkins and Davis proposed a list of musculoskeletal injuries most commonly encountered in tennis players by joint area [31]. Thus, a detailed analysis of MSD risk by region using REBA intermediate scores was proposed to identify the areas most at risk during a slow and fast serve. The ergonomic scores were computed at each moment of the shift by quantified posture analysis using an optoelectronic system.

## 2. Materials and Methods

### 2.1. Participants

Four right-handed young tennis players (17.8 ± 2.2 years, 56.5 ± 4.6 kg, and 1.66 ± 0.08 m) ranked in the first series in the French national ranking voluntarily participated in the experiment. The sample included 2 young males and 2 young females. Detailed characteristics are presented in Table 1. None of them suffered from any joint or muscle injury that might affect serve performance. After a detailed and comprehensive presentation of the entire protocol, each player gave written informed consent before taking part in the experiment. The protocol conformed to the Declaration of Helsinki. The Ethics Committee of the International Institute of Biomechanics and Occupational Ergonomics approved the experiment (IIBOE23-E53).

### 2.2. Experimental Task

Each subject faced a wall 11.88 m away, onto which a tennis net of the required dimensions was projected to reproduce the conditions of a tennis court. The net was surmounted by a target zone to be reached corresponding to a theoretically successful serve. Each player began with a 15 min warm-up session to prevent injury during the experiment. Next, the task was to perform a series of flat serves until five attempts were usable. Each serve was followed by a one-minute rest.

### 2.3. Equipment

After the warm-up session, the players were fitted with 74 markers (14 mm in diameter) positioned all over the body. Fifty-six were anatomical markers positioned on anatomical landmarks identified by palpation in accordance with the recommendations of the International Society of Biomechanics (ISB) [32,33]. Eighteen technical markers were added in clusters of 3 on both arms, forearms, and thighs in order to reconstruct the trajectories of the anatomical markers in the case of occultation. Eight markers were placed around the sieve and on the racket handle to record its position throughout the serve. The markers were carefully positioned so as not to interfere with the racket’s grip [34].

The 3D marker trajectories were recorded using an optoelectronic system comprising ten M5 infrared cameras (Qualisys AB, Göteborg, Sweden) sampled at 150 Hz. A digital camera (Samsung galaxy S20 FE, Samsung Electronics, Seoul, Republic of Korea) was added in the sagittal plane of the player (left) to record each serve entirely and detect the serve key points of interest and the ball’s position.

Two force platforms (600 × 400 mm Kistler 5695A DAQ, Winterthur, Eulachstrasse, Switzerland, 750 Hz) were used to record 3D ground reaction forces (anteroposterior, mediolateral, and vertical axes) throughout the serve. Each subject was asked to start with one foot on each platform and then execute the serve.

### 2.4. Data Processing

Qualisys Track Manager Software (v2020.3 build 6020—Qualisys AB, Gothenburg, Sweden) was used for body tracking and automatic marker labeling. The cubic spline gap-filling function was used to reconstruct anatomical markers in the case of occultation [34,35]. A trial was considered usable if occultations were less than 10 frames. The 20 selected trials (5 trials × 4 subjects) were exported to Matlab (R2023a Update 5, v9.14.0.2237262, The Mathworks, Natick, MA, USA). A Butterworth anti-aliasing low-pass filter (order 2, with a cut-off frequency of 8 Hz) was applied to the data set. The body was modeled in 15 segments as follows: neck, truck, pelvis, left and right arms, forearms, hands, thighs, legs, and feet. An anatomical landmark was defined for each segment at each moment of the serve, based on anatomical markers and in accordance with ISB recommendations. The pelvis was considered the origin of the model, and its 3D position was analyzed in the global reference frame associated with the laboratory with X corresponding to the anteroposterior axis pointing forward, Y corresponding to the vertical axis pointing upwards, and Z corresponding to the mediolateral axis pointing to the right. From this segment, the joint angles of the hips, knees, and ankles, for the lower limbs, and of the neck, trunk, shoulders, elbows, and wrists, for the upper body, were derived from the rotation matrices obtained from the coordinate system of two consecutive segments. The ZXY rotation sequence recommended by ISB was used to compute lower limb joint angles, as well as trunk, neck, elbow, and wrist angles. Only the shoulder sequence was different. Based on recent work, the XZY rotation sequence is preferred to the ISB sequence (YXY), as it is better suited to the analysis of the tennis serve [36].

Twenty-three joint angles were computed at every instant of the serve as follows: neck and trunk flexion (−)/extension (+), left (−)/right (+) inclination and left (+)/right (−) rotation, pelvis anteversion (−)/retroversion (+), left (−)/right (+) inclination and left (+)/right (−) rotation, shoulder and hip flexion (+)/extension (−), abduction (−)/adduction (+) and medial (+)/lateral (−) rotation, elbow flexion (+) and knee flexion (−), forearm pronation (+)/supination (−) wrist flexion (+)/extension (−) and radio (−)/ulnar (+) deviation, and ankle flexion (+)/extension (−).

MSD risk assessment was carried out using the REBA [26]. The proposed grid detailed by Raman et al. was used to compute the REBA score between 1 and 12 [37] (see Appendix A). REBA has the following 5 risk levels: 1 = negligible risk, no action required; 2–3 = low risk, change may be needed; 4–7 = medium risk, further investigation, change soon; 8–10 = high risk, investigate and implement change; 11–12 = very high risk, implement change. A specific script was developed with Matlab to compute the intermediate scores and the final REBA score at each instant of the serve.

Six intermediate scores were successively considered as follows: neck, trunk, leg, upper arm, lower arm, and wrist scores. Joint angle values and specific parameters were used to obtain these six scores. Vertical reaction forces were used to identify take-off and landing instants and thus, the number of ground supports. This information was used to compute the leg score. The force/load score was set to 0, as the weight of a tennis racket is well under 5 kg. The coupling score was also set to 0 because the racket is handled with a power grip. Finally, the activity score was set to 1 because the serve is a fast action with a wide range of changes in posture. All this information was then used to read the intermediate scores needed to determine the final REBA score.

The temporal evolution of the REBA score and each intermediate score was analyzed to determine which joint areas were most at risk and, therefore, which pathologies were likely to appear as a result of repeated service. These data were coupled with the time course of the corresponding kinematic variables for slow and fast services.

**Figure 1:**
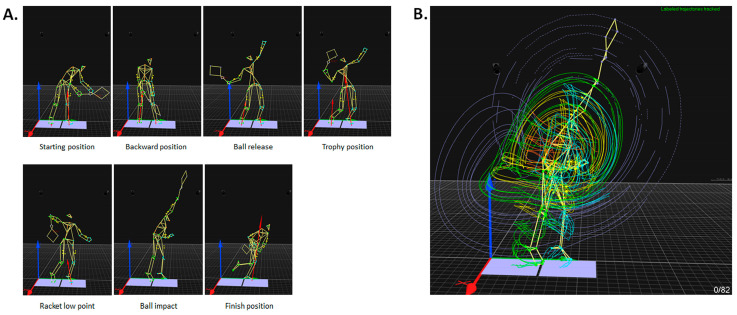
Three-dimensional visualization of markers positioned on players. (**A**) Position of markers at each key point of interest. (**B**) Marker trajectories during the serve. The illustration shows the first attempt by the first male player. The green and blue markers represent anatomical points on the right and left sides respectively. Yellow markers represent technical markers. The purple markers relate to the racket. The two blue squares represent the two force plates. The red vertical arrows represent ground reaction forces.

**Figure 2:**
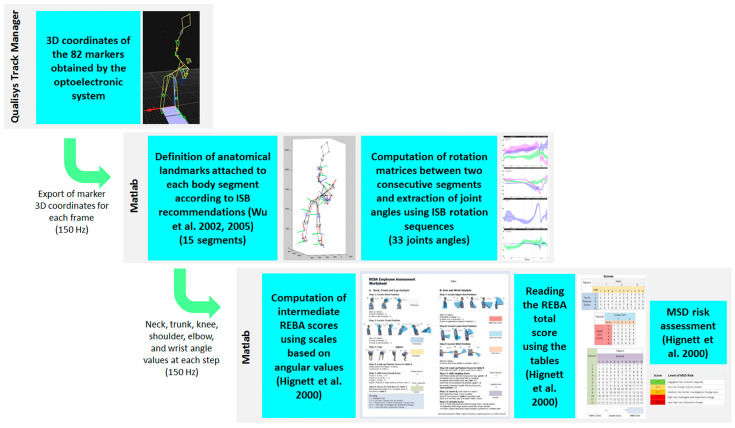
Presentation of the experimental data processing used to carry out an ergonomic assessment of MSD risks during the tennis serve using REBA.

Seven key points were selected to analyze the serve. Five of them, i.e., start, BR, TP, RLP, and BI, were defined as presented in the literature [1,2] and characterized using 3D anatomical marker data. Only BI was identified using the camera previously synchronized with the optoelectronic system. The following key points were added: (1) finish, which corresponds to the end of racket displacement that follows BI before the preparation movement for the next stroke, as presented by Kovacs et al. [1], and (2) backward, which corresponds to the moment when the player’s center of gravity reaches its greatest backward position (smallest value along the X axis of the laboratory global reference frame). The different stages of the serve were defined based on the following 7 key points: release backward between start and backward, release forward between backward and BR, loading between BR and TP, cocking between TP and RLP, acceleration between RLP and BI, and follow through between BI and finish. This division enabled us to study the temporal course of the tennis serve. Figure 1 illustrates the position of the 82 markers for each key point and their trajectory during the serve. Figure 2 shows the entire process of data analysis and ergonomic evaluation using REBA.

### 2.5. Statistical Analysis

A descriptive analysis (mean ± standard deviation) of intermediate and total REBA scores and the corresponding temporal assessment of joint angle was performed for the entire tennis serve. A repeated-measures ANOVA was performed to compare total REBA scores at each key point, taking into account all 20 serves (Statistica 7.1, Statsoft, Tulsa, OK, USA). The significance level was set at 5%.

## 3. Results

### 3.1. MSD Assessment: REBA Analysis

**Figure 3:**
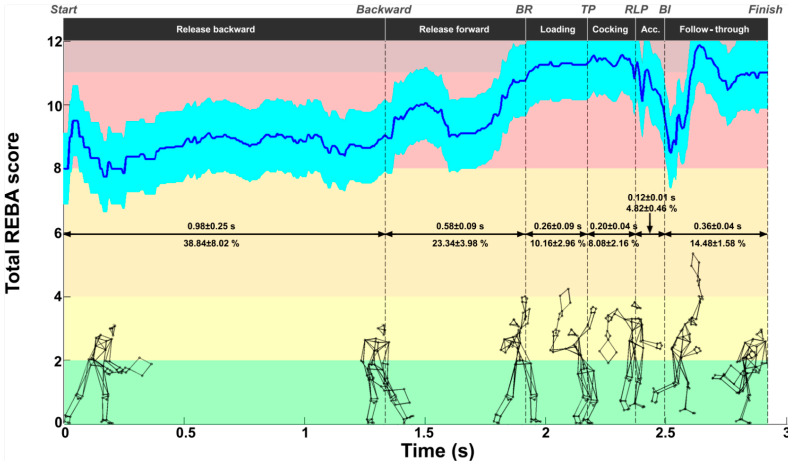
Evolution of the REBA score (mean ± standard deviation) during the tennis serve. The background colors represent the REBA risk level (see last part of Figure 2).

The mean REBA score was 9.7 ± 1.1. Figure 3 shows the evolution of this score during the serve. The average REBA score ranged from 7.7 to 11.8. The lowest scores were observed during the release backward phase. From the second half of the release forward phase, the score increased beyond 11 and was maintained during the loading and cocking phases. A decrease was observed just after BI, followed by an increase to a value close to 11 during the follow-through phase. These scores indicate that the tennis serve presents a high or very high risk with the need to implement changes from an ergonomic point of view. Table 2 presents the REBA score for each serve of each player as well as the mean score for each of the seven key points considered. The highest scores were recorded for TP, RLP, and finish (11.5 ± 0.6, 11.2 ± 0.9, and 11.0 ± 0.3, respectively, *p* < 0.05), while the lowest scores were observed for start, backward, and BI (8.6 ± 1.8, 8.9 ± 0.9, 9.6 ± 1.0, respectively, *p* < 0.05).

The following section presents the REBA results by joint area, according to the REBA evaluation grid: neck, trunk, leg, shoulder, elbow, and wrist.

**Figure 4:**
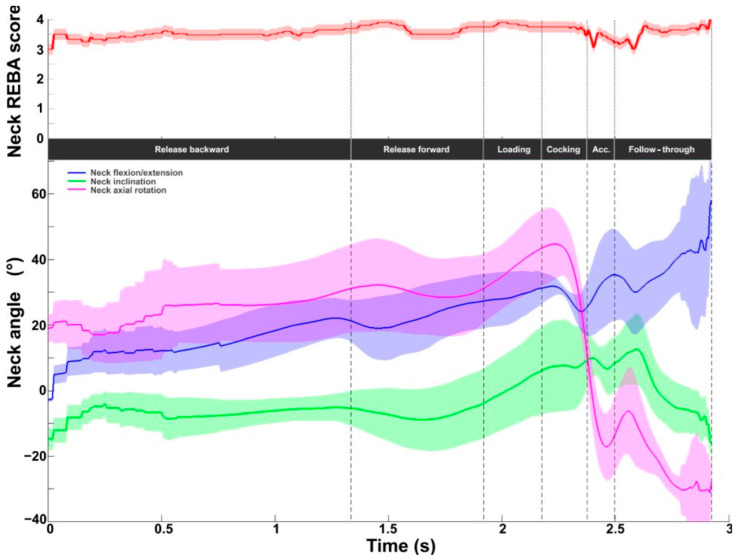
Neck kinematic and ergonomic evaluations during the tennis serve. **Top panel**: Mean (±standard deviation) intermediate neck REBA score. **Bottom panel**: Mean (±standard deviation) neck flexion/extension, inclination, and axial rotation.

The neck score ranges from 1 to 4. The mean value obtained during the serve was 3.6 ± 0.2, with values ranging from 3 to 4 throughout the cycle (Figure 4). Peak values were observed during the loading and the follow-through phases. The lowest values were found around BI. Kinematic evaluation showed that neck extension increased throughout the serve (0° to 60°). Contralateral axial rotation (on the left for right-handed players) increased from 20° to 45° during release and loading phases, with a maximum at the start of the cocking phase, then became zero at RLP and increased again in homolateral rotation (on the right for right-handed players) to 30° at the end of the follow-through phase. Neck inclination averaged between −10° and 10°.

**Figure 5:**
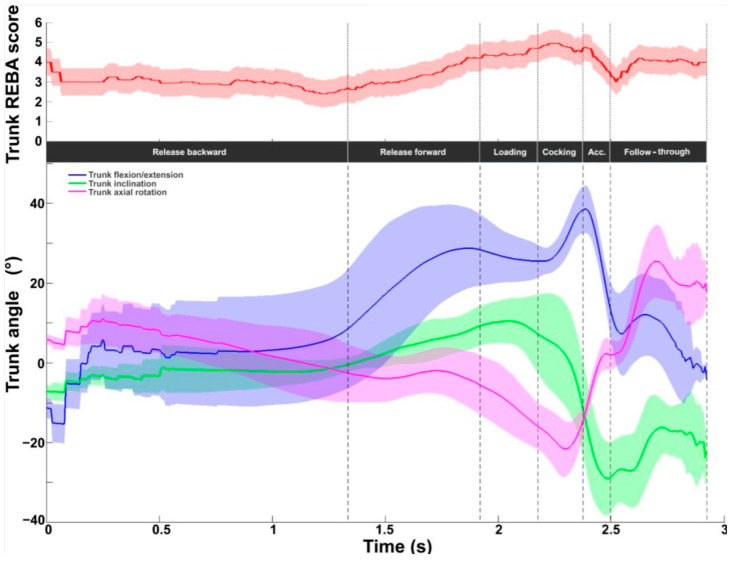
Trunk kinematic and ergonomic evaluations during the tennis serve. **Top panel**: Mean (±standard deviation) intermediate trunk REBA score. **Bottom panel**: Mean (±standard deviation) trunk flexion/extension, inclination, and axial rotation.

Figure 5 depicts the intermediate trunk REBA score evaluated between 1 and 6. The mean value was 3.5 ± 0.7, with values ranging from 2.4 to 5.0. The lowest values were found during the release backward phase. The values then increased to a peak value of 5 during the cocking phase. The values dropped to 3 at BI, then increased to around 4 during the follow-through phase. Trunk extension increased from the beginning to its peak value at RLP (38.4 ± 5.9°) and then decreased to zero. Axial rotation increased on the homo-lateral side (right for right-handed players) to reach a peak value in the middle of the cocking phase (−21.6 ± 7.0°). A rapid rotation to the contralateral side was generated during the acceleration phase, reaching a peak after BI at 25.4 ± 9.2°. The inclination remained between −10° and 10° from the start of the serve to the end of the loading phase, then rapidly increased on the opposite side of the racket during the cocking and acceleration phases, with a peak value at BI (−29.0 ± 9.3°).

**Figure 6:**
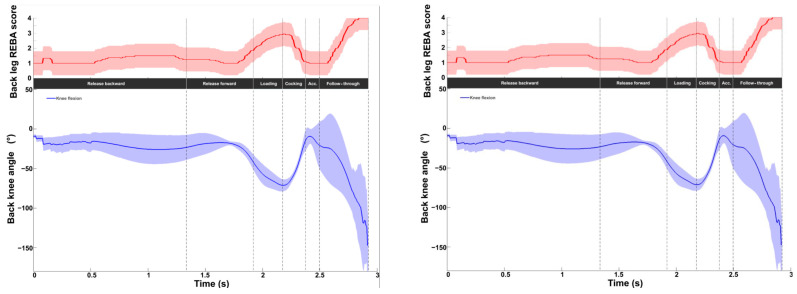
Knee kinematic and ergonomic evaluations during the tennis serve. **Top panels**: Mean (±standard deviation) intermediate leg REBA score. **Bottom panels**: Mean (±standard deviation) knee flexion.

Figure 6 displays the intermediate REBA scores for both knees on a scale of 1 to 4. The values ranged from 1 to 4, with a mean value of 1.4 ± 0.6 for the front knee and 1.6 ± 0.8 for the back knee. A peak value was observed at TP (front knee: 3.0 ± 0.8; back knee: 2.9 ± 0.6). A second peak was observed at the end of the movement, with a higher mean value for the back knee (4.0 ± 0.8 vs. 3.0 ± 0.6). Increases in the scores were directly related to knee flexion. The peak value at the end of the serve for the back knee corresponds to significant flexion (−146.1 ± 32.7°).

**Figure 7:**
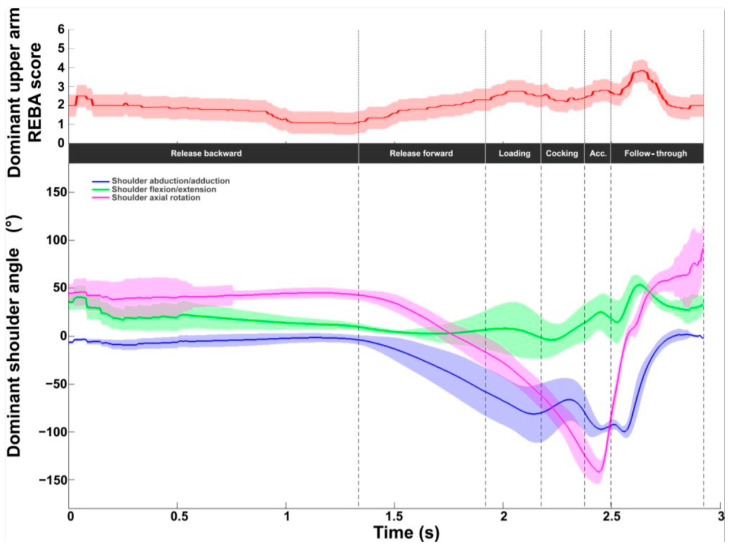
Shoulder kinematic and ergonomic evaluations during the tennis serve. **Top panel**: Mean (±standard deviation) intermediate shoulder REBA score. **Bottom panel**: Mean (±standard deviation) shoulder abduction/adduction, flexion/extension, and axial rotation.

The REBA score for the intermediate shoulder (score between 1 and 6) is displayed in Figure 7. The values ranged from 1 to 4, with a mean value of 2.0 ± 0.6. The values were below 2 during the release backward phase and then increased to reach a maximum value of 3.8 ± 0.6 after BI. The values returned to 2 at the end of the follow-up phase. With regard to kinematics, a constant medial rotation of around 45° and a decrease (40° to 10°) in flexion were observed during the backward release phase. Abduction was zero during this phase. Axial rotation presented a wide angular variation. Lateral rotation increased sharply to reach a maximum lateral shoulder rotation of −141.5 ± 12.6° between RLP and BI (acceleration phase) followed by significant medial rotation until the end of the serve (maximum shoulder medial rotation of 91.9 ± 20.6°).

Abduction increased smoothly during the release forward and loading phases and then slightly before BI (peak value: −99.7 ± 5.7°). Abduction decreased during the follow-up phase, reaching values close to zero. As with flexion, values increased during the cocking and acceleration phases, with a peak during the follow-up phase (53.3 ± 9.0°), then decreased slightly until the end of serve.

**Figure 8:**
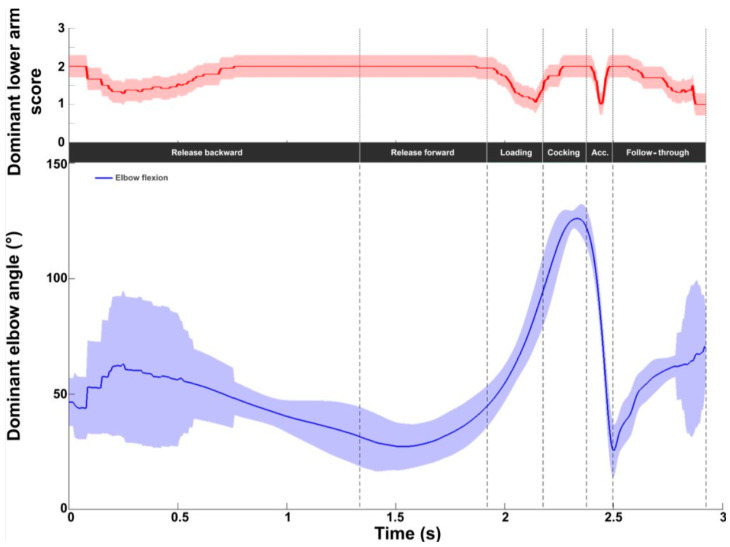
Elbow kinematic and ergonomic evaluations during the tennis serve. **Top panel**: Mean (±standard deviation) intermediate elbow REBA score. **Bottom panel**: Mean (±standard deviation) elbow flexion.

At the elbow, the average intermediate REBA score was 1.8 ± 0.3, with values ranging from 1 to 2 (on a scale of 1 to 3, Figure 8). A score of 2 was observed throughout the release and cocking phases. The lowest values (close to 1) were obtained during the loading and acceleration phases and at the end of the follow-through phase. Flexion values began at around 50°, decreasing during release forward. From halfway through release forward, the flexion values increased to reach a peak during the cocking phase (just before RLP). A sharp decrease was observed during the acceleration phase, with a minimum of 25.6 ± 11.1° at BI, then flexion increased during the follow-through phase.

**Figure 9:**
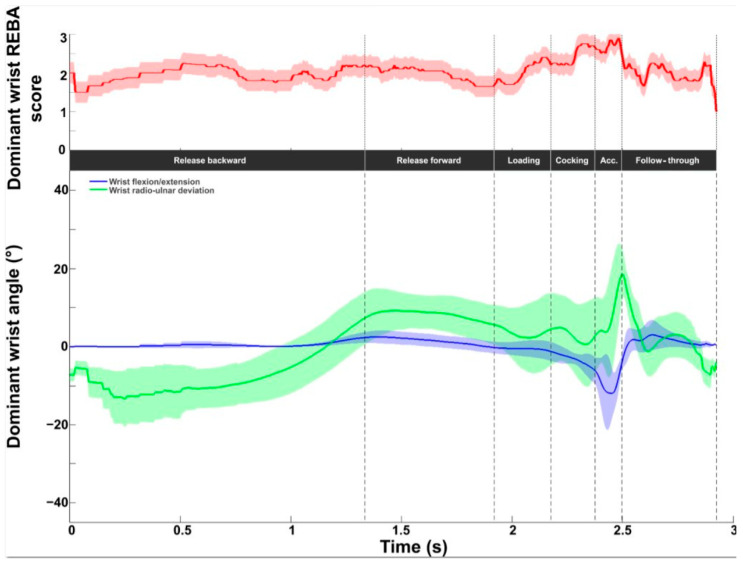
Wrist kinematic and ergonomic evaluations during the tennis serve. **Top panel**: Mean (±standard deviation) intermediate wrist REBA score. **Bottom panel**: Mean (±standard deviation) wrist flexion/extension and radioulnar deviation.

Figure 9 depicts the wrist intermediate REBA score. The values obtained during the serve averaged 2.0 ± 0.3 and covered the whole scale (between 1 and 3). The lowest values were found at the beginning and end. The maximum values were observed in the second half of the cocking phase and the acceleration phase, with a peak value of 2.9 ± 0.3 close to BI. In terms of kinematics, wrist flexion remained close to neutral until the end of the loading phase. Extension increased during the acceleration phase, with a peak value of −12.0 ± 6.9°. After BI, a wrist flexion of around 5° was recorded. A radial deviation was observed in the first phase. From release forward, an ulnar deviation was present until the follow-through phase with a peak of 18.5 ± 7.0° at BI. The RUD remained close to neutral during this last phase.

### 3.2. Performance and Prevention: Slow vs. Fast Serves

**Figure 10:**
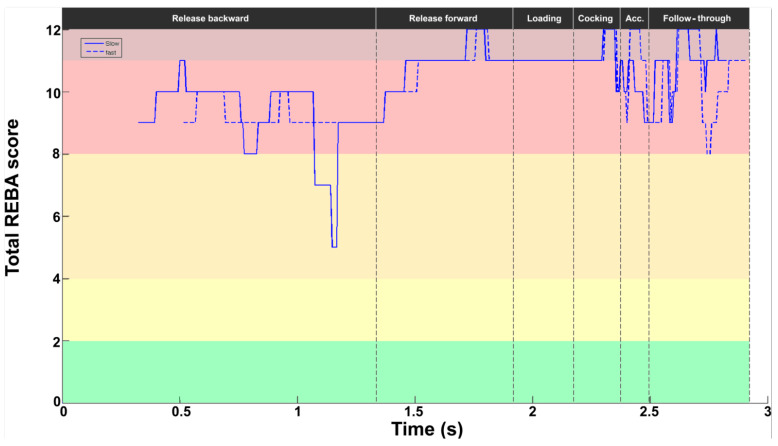
Evolution of the REBA score for slow (solid line) and fast (dotted line) serves. The background colors represent the REBA risk level (see last part of Figure 2).

The REBA score profile was similar between the two serves, with some slight shifts in some stages (Figure 10). The respective REBA scores were 8.8 ± 3.7 and 8.4 ± 4.0 for slow and fast serves. It should be noted, however, that for the slow serve, the score fell sharply during release backward (REBA score of 5). In the acceleration stage, the fast serve showed higher values (peak value at 12), with a reduction in the value up to BI delayed compared with the slow serve. A difference also appeared in the middle of the follow-through stage, with a lower value for the fast serve (8 vs. 11).

**Figure 11:**
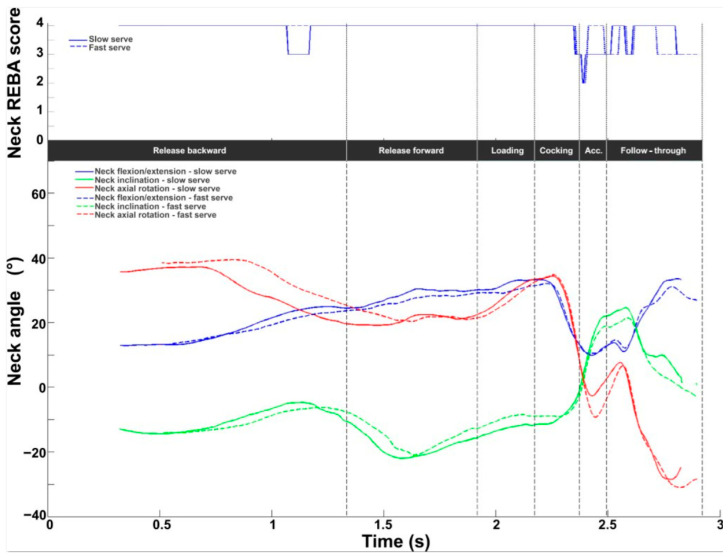
Neck kinematic and ergonomic evaluations for the slow (solid line) and fast (dotted line) serves. **Top panel**: Neck REBA score. **Bottom panel**: Neck flexion/extension (blue), inclination (green), and axial rotation (red).

**Figure 12:**
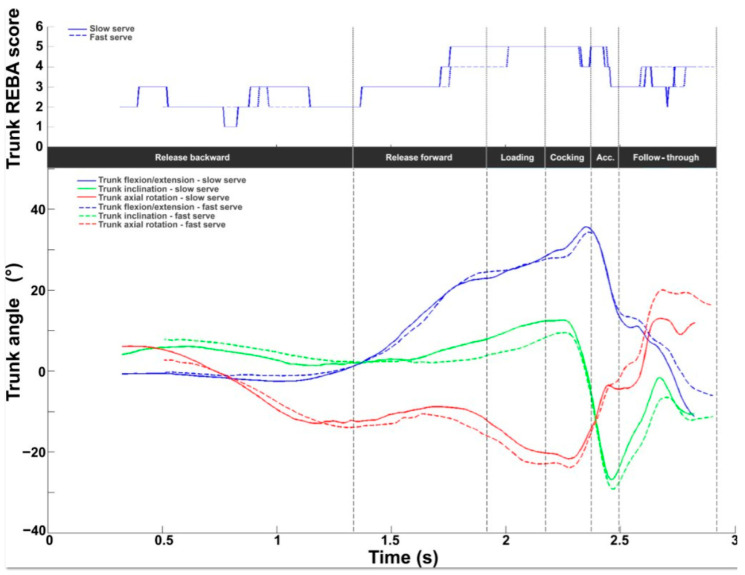
Trunk kinematic and ergonomic evaluations for the slow (solid line) and fast (dotted line) serves. **Top panel**: Trunk REBA score. **Bottom panel**: Trunk flexion/extension (blue), inclination (green), and axial rotation (red).

**Figure 13:**
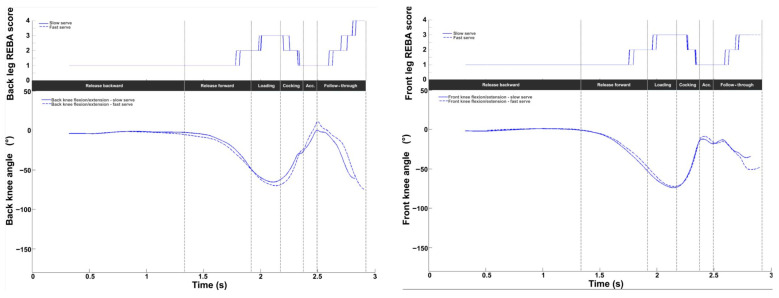
Back (left panels) and front (right panels) knee kinematic and ergonomic evaluations for the slow (solid line) and fast (dotted line) serves. **Top panels**: Knee REBA scores. **Bottom panels**: Knee flexion/extension (blue).

**Figure 14:**
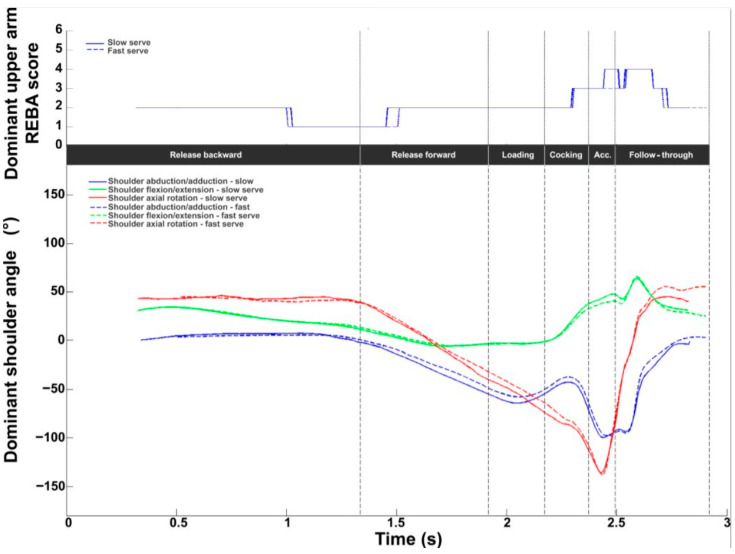
Dominant shoulder kinematic and ergonomic evaluations for the slow (solid line) and fast (dotted line) serves. **Top panel**: Dominant shoulder REBA score. **Bottom panel**: Dominant shoulder abduction/adduction (blue), flexion/extension (green), and axial rotation (red).

**Figure 15:**
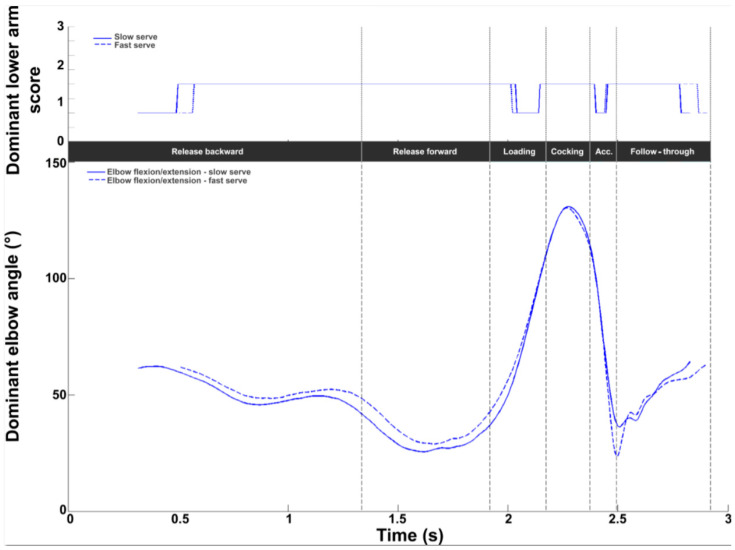
Dominant elbow kinematic and ergonomic evaluations for the slow (solid line) and fast (dotted line) serves. **Top panel**: Dominant elbow REBA score. **Bottom panel**: Dominant elbow flexion/extension (blue).

**Figure 16:**
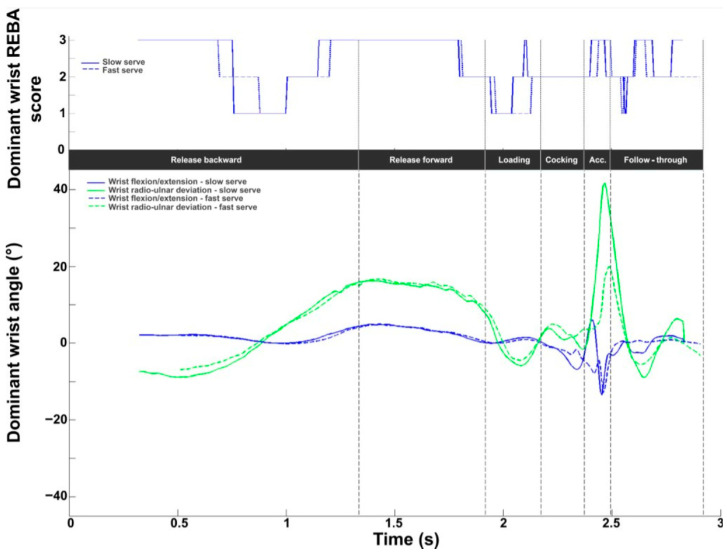
Dominant wrist kinematic and ergonomic evaluations for the slow (solid line) and fast (dotted line) serve. **Top panel**: Dominant wrist REBA score. **Bottom panel**: Dominant wrist flexion/extension (blue), and radioulnar deviation (green).

Figure 11, Figure 12, Figure 13, Figure 14, Figure 15 and Figure 16 show the intermediate ergonomic scores for each joint area in the REBA and the corresponding joint angles for the slow (solid line) and fast (dotted line) serves.

For the neck, the REBA profile presented two differences between the slow and fast serves. During the release backward stage, the score remained constant at 4 for the fast serve, while the slow serve dropped from 4 to 3 for a brief moment. During the following three stages, the profiles remained identical, with scores of 4. During the acceleration stage, the REBA score dropped to 2 at the start and then rose to 4 for the fast serve but only to 3 for the slow serve. Finally, in the follow-through stage, the profile was identical (the score oscillated between 3 and 4), but with a time lag. Regarding joint angles, the profiles were the same for flexion/extension, inclination, and axial rotation. It is interesting to note, however, that rotation was greater for the fast serve during the release and acceleration stages (slow: −2.7°; fast: −9.3°).

For the trunk, the REBA score showed an identical profile between the two serves (values oscillated between 1 and 5) with a time lag. With regard to angles, there were no major differences in the three trunk angles. The greatest difference was observed for inclination during loading (slow: 12.4°; fast: 8.3°) and during the follow-through, for flexion (slow: −11.4°; fast: −6.1°) and rotation (slow: 13°; fast: 20°), but with no impact on serve performance.

For the knees, the REBA scores were almost the same for both knees, with values varying between 1 and 4 for the back knee and 1 and 3 for the front knee. For flexion, no significant difference was observed in the front knee. On the other hand, for the back knee, flexion was slightly greater at TP and lower at BI.

The REBA score profiles for the shoulder were very similar. The values ranged from 1 to 4. A difference was observed at BI. The score was lower (3 vs. 4) for the fast serve. The shoulder profiles in all three planes were very similar between the slow and fast serves.

For the elbow, no difference was observed in the REBA scores between the two serves. A difference of 12.6° was observed at BI. The elbow was less flexed for the fast serve (slow: 36.1°; fast: 23.7°).

For the wrist, the REBA profiles remained close (the values oscillated between 1 and 3), with some shifts in the different phases. With regard to joint angles, significant differences were observed during acceleration. Wrist extension was lower and ulnar deviation was greater during the slow serve (slow: 41.7°; fast: 20.0°).

Table 3 summarizes the differences observed for one player in terms of kinematic variables and the associated risks of MSD occurrence. This information could subsequently be used by coaches or trainers to link MSD prevention and performance.

## 4. Discussion

The aim of this study was to evaluate tennis serve performance by considering the risk of MSD incurred by a player with regard to posture and the characteristics of the task, using the REBA tool. To address this original challenge, which has never been considered in the literature, a slow serve and a fast serve were compared. For this purpose, a 3D kinematic analysis of the serve was carried out. The body was modeled using 15 segments. Their displacements and relative joint angles were computed at each instant to obtain an evolution over time. The serves were divided into six stages using seven key points classically identified in the literature. Two force platforms were used to identify the flight phase and the number of feet on the ground during the support phase. All these data were used to quantify six intermediate REBA scores for six joint areas (neck, trunk, leg, shoulder, elbow, and wrist), as well as the total score reflecting the level of risk of MSD occurrence throughout the serve.

### 4.1. Tennis Serve and MSD Risk

Ergonomic analysis of the tennis serve revealed an average REBA score of 9.7 ± 1.1 across all stages, corresponding to “high-risk activity”. Loading, cocking, and follow-through are the highest risk stages, with mean scores above 11, i.e., “very high-risk activity” [26]. This first result is in line with the literature and the number of injuries identified in tennis. The main injuries that affect the musculoskeletal system reported are as follows: shoulder (rotator cuff inflammation [38]), elbow (medial or lateral epicondylitis, i.e., tennis elbow [39]), wrist (tendonitis, e.g., De Quervain’s tenosynovitis [40]), back (low back pain [41] due to lumbar disc degeneration and herniation [42]), knee (tendonitis, bursitis or meniscal lesion [43]), and ankle (sprain, plantar fasciitis or Achilles tendonitis [44]).

The temporal analysis proposed in this study highlighted the areas most exposed to MSD in relation to the six REBA joint areas, as well as the times when they were most exposed. For the neck, the REBA score was between 3 and 4 (out of 4), indicating a highly exposed area throughout the serve. The neck was continuously in rotation (+20° left or right) and in increasing extension throughout the six stages of the serve. These postures are the cause of a high intermediate ergonomic score throughout the serve. However, the risk level could be modulated. Indeed, during the release and cocking stages, movements are controlled and executed slowly, which would considerably reduce the risk of injury, according to Lee’s study [45]. Conversely, fast neck rotation in extension during the second part of cocking and acceleration stages increases compressive and torsional stresses on spinal vertebrae and predisposes the neck to injury of an acute or chronic nature. In extension and rotation, the diameter of the intervertebra foramina through which nerve roots pass is decreased [46]. High ballistic, rotational forces passing through this area predispose the right zygapophysial joints and surrounding nerve and soft tissue to trauma [45]. The neck is therefore an area at risk of TMS because of its constant extension to maintain visual contact with the ball and the quick rotations caused by the high intermediate REBA score. Therefore, it is necessary to be aware of this joint, even if it has been considered not to be the most exposed area, especially considering the large number of serve repetitions in training and during matches in a year.

For the trunk, the intermediate score was between 2.5 and 5 (out of 6). The highest scores were observed for the loading and cocking stages (>4/6). With the exception of the follow-through stage, the trunk was in extension, with a value that increased from release to TP, where the peak value appeared. These values are in line with other studies on the trunk during the tennis serve [19,47]. This posture is already associated with the presence of an MSD risk in ergonomic tools [26,27,28]. On the other hand, during these two stages, the trunk was also rotated and inclined, which increased the risk of MSD with scores of 5/6. This usually translates into lower back pain associated with lumbar strain. The pain is partly muscular, involving the extensors, flexors, and rotators of the spine (multifidus).

The main cause would be alternating concentric/excentric contraction of these muscle groups to go from an extreme extension rotation to extreme flexion rotation during the serve [42]. These combined movements induce greater stress on the vertebrae than movements in a single plane, thus increasing the risk of pain and injury [48]. Moreover, as shown by Campbell et al. in elite adolescent tennis players, lumbar joint reaction moments during the acceleration phase (3 to 40 times greater than running) highlight the “high” loading conditions of the lumbar region, which could be at the origin of the development of low back pain during the repeated tennis serve [49].

For the shoulder, the intermediate REBA score was 2.0 ± 0.6 (on a scale of 1 to 6), reaching a peak of 3.8 ± 0.6 during the follow-through stage. This result does not directly indicate a significant risk of MSD during the serve. However, several studies have reported numerous shoulder injuries in tennis players. The main cause would be the large joint ranges in lateral rotation (−141.5 ± 12.6° in agreement with other studies [20,50]) and the high medial rotation velocities generated in the acceleration stage [19,21]. This overloading of the joints and muscles of the shoulder girdle would lead to inflammation of muscular tendons (biceps brachii and rotator cuff muscles) or joints (bursitis) or to deterioration of shoulder joint structures such as the ligament capsule or labrum [42,51,52]. This disparity between the low REBA intermediate score for the shoulder and the fact that the tennis serve is the cause of many injuries highlights the limitations of the REBA tool in sports. Indeed, the specificity and complexity of the serve impose shoulder motions that are not taken into account in the assessment of the intermediate score. The REBA assessment mainly dichotomizes the shoulder flexion–extension motion (five angular sectors), with a +1 increase in the case of rotation (with no precise value), whereas the tennis serve mainly involves rotational movement, which underestimates the REBA risk assessment.

For the elbow, the mean intermediate REBA score was 1.8 ± 0.3, with values ranging from 1 to 2 (on a scale of 1 to 3). This may translate into an intermediate risk of MSDs. However, as for the shoulder, the elbow is often affected by injuries. The origin can be found in the acceleration stage, where flexion decreases from 125.7 ± 4.7° to 25.6 ± 11.1° in 0.12 ± 0.01 s, which is in line with previous studies by Kibler et al. (extension from 116° to 20° of flexion within 0.21 s [53]) and Fett et al. (from 132.2 ± 10.4° to 18.0 ± 8.5° during acceleration stage [19]). Because of the combined rotation of the shoulder, this results in a double load on the elbow called “valgus extension overload”, the cause of epicondylitis, in particular [54]. Lateral epicondylitis, or “tennis elbow” [55], is most common, affecting an average of one in two players [39,56]. The main causes are poor tennis technique [57], often observed in beginners, large racket size [58], and high repetition of the one-handed backhand [39]. Pain results from microtearing of the extensor carpi radialis brevis [39]. Nowadays, lateral epicondylitis prevalence has decreased because of improved technical outcomes and the two-handed backhand [55].

For the wrist, the intermediate REBA score was 2.0 ± 0.3 (maximum 3). The maximum score was reached during the second half of the cocking phase and the acceleration phase, with a peak value of 2.9 ± 0.3 close to BI. These values show that the maximum risk of MSD occurrence was reached for these stages. These high scores can be explained by a quick flexion movement from an extended position during the acceleration phase (−12.0 ± 6.9° to 4.2 ± 2.5°), coupled with a large ulnar deviation, particularly at BI (18.5 ± 6.9°). Similar values have been reported in recent studies, notably for wrist flexion at BI by Wang et al. (5.3 ± 2.9° for expert players [9]) and Fleisig et al. (15.0 ± 8.0° for men and women [21]). These wrist joint angles are associated with injuries such as extensor carpi ulnaris tendinosis and instability, tenosynovitis, stress fractures, and injuries to the triangular fibrocartilage complex [59], which account for one-third of all upper limb injuries [60]. These harmful postures are exacerbated by internal forces (muscular forces and torques) and external forces (due to the interaction between the ball and the racket at BI) during the stroke [61]. Although torques are probably lower than the levels at which tissues sustain permanent structural damage, repetitive hitting with wrist angular configurations far from joint neutral would favor the development of wrist lesions in tennis players due to overuse [62]. In professional tennis players, over 1000 strokes can be recorded during a match lasting between 3 and 5 h, with several matches played with less than 48 rests during the Grand Slams [63].

Finally, for the knees, intermediate REBA scores were very similar between the front and back knees (front knee: 1.4 ± 0.6; back knee: 1.6 ± 0.8). The scores ranged from 1 to 3 for the front knee and from 1 to 4 (out of 4) for the back knee. The difference was observed during the follow-through stage and corresponded to greater knee flexion for the back knee. The angular variations observed between the moment of greatest knee flexion (TP back knee flexion: −71.3 ± 7.9°; TP front knee flexion: −66.6 ± 4.7°) and that of least flexion (during the acceleration phase, i.e., between RLP and BI, back knee flexion: −9.5 ± 9.3°, front knee flexion: −15.4 ± 5.8°) correspond to the values reported in the literature for these two moments. Several authors have found TP knee flexion values between 60 and 80° for different ages [12], serve types [13], and men and women [11]. Fleisig et al. reported a low front knee flexion of 13.0 ± 8.0° at RLP [21]. Fett et al. [19] and Whiteside et al. [2] found similar values at BI between 5° and 20° for the back knee and between 15° and 30° for the front knee. These data are in line with the values measured in the present article. The knee is a highly solicited joint and ranks among the areas exposed to injury behind the shoulder and back, with a prevalence of around 20% [64]. The most commonly observed pathologies are patellofemoral dysfunction, jumper’s knee, meniscal injuries, and bursitis [43]. These disorders affect the structural elements of the joint, i.e., alignment of the bony and muscular structures of the knee, in particular the extensor muscles, menisci, ligaments, and bursae, and are the result of overuse in flexion and torsion during stance [31,43].

In tennis, the risks are more related to angular variations during jump preparation (loading stage), fast extension during the cocking stage (extension velocities between 450 and 800°/s reported in the literature [19,21,65]), and high loading of the front lower limb joints during jump landing (follow-through stage). It was during these stages that the intermediate REBA scores were highest (>3 out of 4) and, therefore, the risk of MSD would be greatest (TP front knee: 3.0 ± 0.83; TP back knee: 2.9 ± 0.6; follow through front knee: 3.0 ± 0.6; follow through back knee: 4.0 ± 0.8).

The results presented were obtained for a group of young national-level players (17.8 ± 2.2 years). Wang et al. [9] showed that postures were affected by player level at different key points. The authors found differences in the trunk at trophy position and in the whole upper limb at ball impact. Whiteside et al. [2] found an effect of age on posture. A difference in peak trunk inclination was observed during trophy position between a group of children (10.6 ± 0.6 years) and a group of young people (14.8 ± 0.5 years). In a second study, Whiteside et al. [8] also showed an age effect on posture (trunk, pelvis, and upper limb) during trophy position and ball impact. These differences in joint angles as a function of age and level of expertise are important since they modify postures and consequently the results of the REBA ergonomic evaluation. It might therefore be appropriate to reproduce the ergonomic assessment at different ages and different levels to study the evolution of MSD risks during the tennis serve.

### 4.2. MSD Prevention and Performance

The comparison of a slow and fast serve for one player highlighted some differences between the performance achieved and the associated potential MSD risks. The overall assessment showed differences in MSD risk during the acceleration phase, with a total REBA score of at least one point for the fast serve. The following parameters were identified: neck axial rotation and shoulder flexion. For neck rotation, the intermediate REBA score was one point higher for the fast serve and could be linked to a more significant rotation than during the slow serve. On the other hand, for the shoulder, the risk of MSD was one point lower for the fast serve with less flexion than for the slow serve for the same stage.

On the other hand, joint angle differences were observed for elbow flexion and wrist ulnar deviation, but with no impact on the intermediate REBA score. This may be explained by the thresholds chosen at which the risks change. In the literature, it is known that level has an influence on MSD risk. Indeed, poor technique, often observed in beginners, has been associated with a higher risk of injury [31]. In training, this information could be used by coaches, trainers and players to improve performance while reducing MSD risks.

### 4.3. Application of Key Findings

The results presented in this work address our twofold objective to (1) carry out an ergonomic assessment of MSD risks and (2) associate this level of risk with the evaluation of performance during the tennis serve.

The first point proposed the temporal evolution of the kinematic variables of the following six joint areas included in the REBA tool: shoulder, elbow, wrist, neck, trunk, and legs (through the knee joint and the number of supports). Thanks to 3D analysis, all the joint angles of these joints were measured during the entire serve, divided into stages based on key points. This approach is totally original as, to our knowledge, no other study has proposed such a kinematic analysis of the serve. The majority of works proposed values at key instants without a temporal evaluation. The few works that proposed a temporal evaluation only considered a few body areas, such as the knee [3,5], or only the upper limb [66] or lower limb. A recent study investigated 28 joint angles of the upper and lower limbs and proposed a temporal evaluation of the 13 angles correlated with racket velocity, but only for the cocking and acceleration stages [34].

Kinematic evaluation was used to quantify postures in each step. These data were used as input to the REBA tool to qualify and quantify serve-related MSD risks based on posture and general task characteristics that had never been addressed before. Integrating this analysis into the performance analysis highlighted a number of differences that could lead to the medium-term consideration of player protection as part of performance optimization.

The results of our work enabled two ways to be identified. The first would be to modify the serve technique through the kinematic parameters involved in the high REBA score observed, in order to reduce the MSD risk while maintaining equivalent racket velocity. The second would be to propose muscle-strengthening or stretching exercises that would reduce the long-term occurrence of MSD throughout the player’s career, despite the high REBA score (and, therefore, the risks). These two lines of action could be the subject of future research to protect athletes in the course of their sporting activities.

### 4.4. Limitations

Some limitations of this study could be addressed. This study was carried out on five serves per player and only for four players. In fact, this work is difficult to generalize to middle-aged and older tennis players. As the young players studied are not yet international players, the results are also difficult to transfer. Extending the analysis to a larger sample would enable generalizing the proposed results and studying the effect of different parameters (type of serve, stance style, age, expertise) on MSD risk.

The REBA tool is a generic ergonomic tool that was developed primarily for the assessment of work-related postures, with a predominantly analysis-based design. However, the tennis serve is a complex gesture involving numerous rotations in different planes. As a result, some risks are probably underestimated. At present, there are no ergonomic tools linked to the sport, and REBA is the one that takes into account the most elements of the activity in assessing risk. Future work on more suitable tools could be carried out in order to propose an MSD assessment more specific to sports.

## 5. Conclusions

The present study proposed a kinematic analysis of the six major joint areas of the body during the tennis serve. Joint angular evolutions were presented as a function of time for each stage of the serve in relation to the various key points of interest identified in the literature. These full-body kinematic data were used to perform an ergonomic assessment using the REBA tool at each point in time. The results showed that tennis serve is a high-risk activity that varies according to phase, with the greatest risk during the loading and cocking stages. The causes of these risks were expressed with reference to kinematic variations. An analysis of the slow and fast serves and associated performance values was proposed. The REBA profiles were similar, with an average score of 8.8 ± 3.7 and 8.4 ± 4.0, respectively, for the slow and fast serves. The maximum REBA score (12/12) was reached during the acceleration phase. The fast serve showed a one-point increase in the intermediate neck REBA score with an increase in axial rotation of +6.6° and a one-point decrease in the intermediate shoulder REBA score with a reduction in flexion of 7° during the acceleration phase. The data can be used by coaches and athletes to improve performance while trying to prevent the occurrence of MSDs.

## Figures and Tables

**Table 1:** Detailed characteristics of the measured players.

	Player 1	Player 2	Player 3	Player 4	Mean ± Std
Sex	Male	Male	Female	Female	
Age (year)	21	17	17	16	17.8 ± 2.2
Height (m)	1.75	1.74	1.63	1.59	1.66 ± 0.08
Weight (kg)	58.1	61.0	58.8	50.4	56.5 ± 4.6
BMI	19.0	20.2	22.1	19.9	20.3 ± 1.3
Training by week (h)	15.0	15.0	15.0	15.0	15.0 ± 0.0
Level	National	National	National	National	

**Table 2:** REBA score for each serve and each player computed for the 7 key points of interest.

		Start	Backward	BR	TP	RLP	BI	Finish
Player 1	Serve 1	9	9	11	11	10	9	11
	Serve 2	10	9	11	11	11	9	12
	Serve 3	9	9	11	11	11	9	11
	Serve 4	9	9	11	11	11	9	11
	Serve 5	10	9	11	11	11	9	11
Player 2	Serve 1	7	10	11	12	12	7	11
	Serve 2	7	10	7	12	12	10	11
	Serve 3	7	8	8	11	10	10	10
	Serve 4	8	8	7	12	10	10	11
	Serve 5	7	8	7	12	10	10	11
Player 3	Serve 1	11	10	12	12	12	12	11
	Serve 2	12	10	12	11	12	11	11
	Serve 3	11	10	12	10	12	10	11
	Serve 4	8	8	12	12	12	9	11
	Serve 5	10	9	11	11	12	9	11
Player 4	Serve 1	7	8	11	12	11	10	11
	Serve 2	7	10	11	12	11	9	11
	Serve 3	5	8	10	12	9	10	11
	Serve 4	10	8	11	12	12	9	11
	Serve 5	7	8	10	12	12	10	11
	Mean	8.6 ± 1.8 ^3457^	8.9 ± 0.9 ^3457^	10.4 ± 1.7 ^124^	11.5 ± 0.6 ^1236^	11.2 ± 0.9 ^126^	9.6 ± 1.0 ^457^	11.0 ± 0.3 ^126^

^1^ different from start; ^2^ different from backward; ^3^ different from BR; ^4^ different from TP; ^5^ different from RLP; ^6^ different from BI; ^7^ different from finish.

**Table 3:** Kinematic parameters that differ between the slow and fast serves and affect the MSD risk level.

Joint	Stage/Key Point		Comparison Slow vs. Fast
Neck	Acceleration	REBA	+1 for fast serve
		Axial rotation	+ 6.6°
Shoulder	Acceleration	REBA	−1 for fast serve
		Flexion	−7°
Elbow	BI	REBA	NS
		Flexion	−12.5° for fast serve
Wrist	BI	REBA	NS
		Ulnar deviation	−5.5° for fast serve

**Figure A1:**
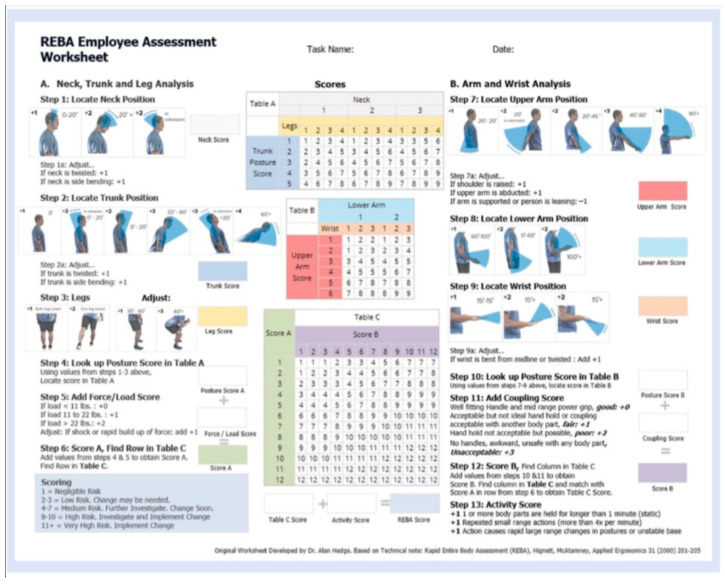
REBA method summary sheet. The left and right columns show the method for computing the intermediate scores, while the middle section contains the conversion charts for obtaining the final REBA score.

## Data Availability

Data are available upon request.

## Appendix A

REBA grid adapted from Hignett et al. [26] for computing MSD risk associated with the tennis serve (extracted from Raman et al. [37]).
